# Understanding Social Network and Support for Older Immigrants in Ontario, Canada: Protocol for a Mixed-Methods Study

**DOI:** 10.2196/12616

**Published:** 2019-03-04

**Authors:** Sepali Guruge, Souraya Sidani, Lu Wang, Bharati Sethi, Denise Spitzer, Margaret Walton-Roberts, Ilene Hyman

**Affiliations:** 1 Ryerson University Toronto, ON Canada; 2 King’s University College London, ON Canada; 3 University of Ottawa Ottawa, ON Canada; 4 Wilfred Laurier University Waterloo, ON Canada; 5 University of Toronto Toronto, ON Canada

**Keywords:** geographic information system, immigrants, social network, social support

## Abstract

**Background:**

Older adults are the fastest growing age group worldwide and in Canada. Immigrants represent a significant proportion of older Canadians. Social isolation is common among older adults and has many negative consequences, including limited community and civic participation, increased income insecurity, and increased risk of elder abuse. Additional factors such as the social, cultural, and economic changes that accompany migration, language differences, racism, and ageism heighten older immigrants’ vulnerability to social isolation.

**Objective:**

This mixed-methods sequential (qualitative-quantitative) study seeks to clarify older immigrants’ social needs, networks, and support and how these shape their capacity, resilience, and independence in aging well in Ontario.

**Methods:**

Theoretically, our research is informed by an intersectionality perspective and an ecological model, allowing us to critically examine the complexity surrounding multiple dimensions of social identity (eg, gender and immigration) and how these interrelate at the micro (individual and family), meso (community), and macro (societal) levels in diverse geographical settings. Methodologically, the project is guided by a collaborative, community-based, mixed-methods approach to engaging a range of stakeholders in Toronto, Ottawa, Waterloo, and London in generating knowledge. The 4 settings were strategically chosen for their diversity in the level of urbanization, size of community, and the number of immigrants and immigrant-serving organizations. Interviews will be conducted in Arabic, Mandarin, and Spanish with older women, older men, family members, community leaders, and service providers. The study protocol has received ethics approval from the 4 participating universities.

**Results:**

Quantitative and qualitative data collection is ongoing. The project is funded by the Social Sciences and Humanities Council of Canada.

**Conclusions:**

Comparative analyses of qualitative and quantitative data within and across sites will provide insights about common and unique factors that contribute to the well-being of older immigrants in different regions of Ontario. Given the comprehensive approach to incorporating local knowledge and expert contributions from multilevel stakeholders, the empirical and theoretical findings will be highly relevant to our community partners, help facilitate practice change, and improve the well-being of older men and women in immigrant communities.

**International Registered Report Identifier (IRRID):**

DERR1-10.2196/12616

## Introduction

### Background

Older adults (aged 65 years or older) are the fastest growing age group worldwide, and in Canada, they are projected to comprise 25% of the population by 2050 [1]; thus, maintaining their well-being is a priority in Ontario, Canada [2]. Older immigrants form a large proportion of the older adult population in Canada, with the highest percentage of older immigrants residing in Ontario [3]. Older immigrants contribute to society through paid work and unpaid labor (childcare, cooking, and cleaning) that reinforces the overall economic well-being of the family and supports the educational pursuits and labor market activities of children and grandchildren. In addition, they often participate in volunteerism that promotes community cohesion and the development of social capital [4].

Multiple factors (eg, death of family or friends, retirement, and health or mobility problems) can negatively affect their active family and community engagement, which can potentially lead to their social isolation [5-8]. Social isolation, in turn, can negatively affect their income security, vulnerability and ability to respond to elder abuse, access to services and supports, and overall quality of life and ability to age well, independently and with dignity. Evidence suggests that social isolation is a risk to mental health [9-11].

Social support, “the interactive process in which emotional, instrumental, or financial aid is obtained from one’s social network” [12], is key to promoting well-being among older adults [13,14]. Social networks can be understood as “the web of identified social relationships that surround an individual and the characteristics of those linkages... It is the set of people with whom one maintains contact and has some form of social bond” [12]. Informal social networks can include family, friends, neighbors, and coworkers [15]. Newcomers (<10 years in Canada) often experience a significant loss in the quantity and quality of social networks and support [16-21]. The quantity and the quality of social networks and support can affect access to employment, transportation, food, and accommodation. Social support can ease the settlement and integration process [10,22] and promote resilience and capacity in the postmigration context by enhancing a sense of belonging, reducing exposure to and the effects of racism, and improving access to information and services [22-32]. Support from people within a shared ethnic community is often associated with better settlement and integration outcomes [22]. Ethnic communities can provide opportunities for religious participation, economic activity, and engaging in familiar roles and statuses, which can encourage political mobilization and material opportunities and reduce the effects of discrimination and racism [33]. However, there is also research that suggests living in too close proximity to ethnic neighborhoods may have adverse effects. Ethnic density in large urban settings can be the result of discriminatory housing policy, economic restructuring, and other external constraints on individuals, families, and communities and may not always have a positive impact on individuals [34-36]. Smaller ethnic communities, especially in smaller cities, may permit stronger social bonds that promote well-being [37]; they may also be perceived as less of a *threat* by the dominant group and, therefore, treated more positively.

Informal social networks can also be sources of conflict and abuse [9,22,38] or lead to pressure for reciprocity [39], with negative effects on the well-being of older immigrants [40]. For example, abuse of older immigrant women by family members has significant negative effects on their well-being, which can be exacerbated by ethnic community control of the older adults’ decisions about leaving the abuser, living alone, or engaging in paid employment [16]. As the sizes of the informal social networks vary across situations and contexts, formal sources of support may play an important role in helping older immigrants. Formal social support may be provided, for example, by social service or settlement agencies, health clinics, legal clinics, or police officers [15]. Successful settlement and integration are dependent on the quantity and quality of services that meet the needs of immigrants [41-43]. Funding cuts over the last 10 years, especially for new and sustainable programs, have reduced the quality and availability of services for immigrants [44,45]. In addition, immigrants, especially some subgroups (eg, older men and older women), underutilize shelters; hotlines; and social, health, and legal services [46-54] because of multiple intersecting factors such as lack of familiarity with services; lack of linguistically appropriate services; lack of accessible, portable, and coordinated services; confidentiality concerns; as well as discriminatory and racist practices embedded within services and service delivery [49,55].

Currently, programs and initiatives supporting older adults are often separate from those supporting immigrants and involve different ministries, organizations, and levels of government, which has resulted in a lack of cohesion among formal supports and a lack of awareness about what is available to older adults across organizations and sectors. Older immigrants are often more socially isolated than their nonimmigrant peers and face additional challenges, including language barriers and lack of familiarity with the new postmigration spaces and places [26,38]. Little is known about how older immigrants access local social networks or how these differ by their sociodemographic characteristics and by the size of the city in which they reside. Furthermore, access to and use of social (formal and informal) networks have not been compared and contrasted across large, medium, and small cities.

### Study Objectives

The study objectives are to (1) understand the social needs, the nature and composition of networks, and the availability of social supports for older immigrants in Ontario as perceived by older men and women, family members, and leaders belonging to long-term and established (Chinese) and relatively newcomer and less-established (Middle Eastern and Latin American) immigrant communities and service providers and (2) identify the factors affecting older immigrants’ access to and use of informal and formal social supports, geographic inequalities, and the gaps between service needs and provision and how these shape older immigrants’ capacity, resilience, and independence.

## Methods

### Design

The study consists of a mixed-methods, sequential (qualitative-quantitative) design, conducted in 2 phases. Consistent with the collaborative, community-based approach to research, stakeholders representing the different immigrant communities and potential participants (eg, older adults, family members, and community leaders) have been involved in the development of the study proposal and in the formation of an advisory committee. The latter committee will provide recommendations on the recruitment of participants, data collection, and interpretation of the findings to ensure that our research process is culturally responsive and that the outcomes and outputs are relevant to stakeholders.

Phase 1 uses a qualitative approach to explore older immigrants’ actual or preferred size and composition of informal and formal networks, type, model of delivery (eg, use of technology) and frequency of support used or preferred or accessed, and perceptions of reciprocity and conflict within such networks. Phase 2 is primarily quantitative and aims to clarify the gaps between older immigrants’ needs and available services.

### Setting

This study will be conducted in 4 cities in Ontario: Toronto, Ottawa, Waterloo, and London. These sites have been chosen to compare across large, medium, and small cities. Three language groups (Arabic, Mandarin, and Spanish) were selected for the size of the particular language-speaking population in these cities and based on the advice and feedback from our community advisory committees at each of the 4 sites.

### Participant Recruitment

The inclusion criteria for older women and men are as follows: aged 60 years or older; have been living in Canada for less than 20 years; currently residing in 1 of the 4 cities (London, Ottawa, Toronto, or Waterloo); and speak Arabic (if in London, Ottawa, or Toronto), Mandarin (if in Toronto or Waterloo), or Spanish (in any of the 4 cities) as a first or primary language. Family members will be selected if they are aged 18 years or older and have 1 or more parents or grandparents who meet the study inclusion criteria.

We will recruit community leaders and service providers using our existing networks and contacts. The inclusion criteria for community leaders are as follows: aged 18 years or older and self-identify as a member of the selected immigrant communities and a leader (eg, educator, religious leader, legal advisor, politician, or advocate) in the community who works with older women and men living in the respective city. Service providers will be selected if they are aged 18 years or older and, in a paid (employment) capacity, provide services (such as educational, health, legal, or social services) to older immigrants belonging to the selected communities.

*Phase 1* will involve data collection with 5 stakeholder groups: older women, older men, family members, community leaders, and service providers. We will explore the social support needs of older immigrants as well as the nature and composition of actual or preferred informal and formal networks. Given the diversity of older immigrant population with respect to location, size, and the availability of community-specific networks and resources, (as mentioned earlier) we will focus on Spanish, Mandarin, and Arabic in Toronto; Spanish and Arabic in Ottawa; Spanish and Arabic in London; and Spanish and Mandarin in Waterloo. Recruitment of participants will take place primarily via referral through the connections we have with the respective immigrant communities. We found this recruitment method to be effective in our previous research involving immigrant communities [15,56].

Data collection will be done through focus group sessions. These discussions allow participants to respond to each other’s comments; to question, clarify, and elaborate on ideas; and to reach consensus about collective knowledge [57] within a short period. Focus group sessions will (1) follow a comparable protocol (combination of open-ended and standardized questions) that allows flexibility and evolution over the course of the study in response to emerging findings, (2) be held at participants’ convenience and in the language of their choice and audio-recorded (with consent), (3) be cofacilitated by trained research assistants who speak the primary language of the community and/or have experience working with the particular community, and (4) be offered (as much as possible) at different locations in each city to make it convenient for participants to attend. Data collection sessions will be held separately with each of the 5 stakeholder groups at each city. For older women and older men, we will conduct separate sessions by gender to maximize comfort and encourage dialogue. Our previous work on sensitive topics (eg, abuse and violence) has shown that this type of group setting can create a safe environment in which self-selected women and men from immigrant communities discuss topics of importance to the participants quite openly. The group discussion will focus on exploring the social needs of older immigrant women and men, describing the nature and composition of their actual and preferred social networks, and identifying factors that contribute to their access to formal and informal supports within their community and area of residence.

Purposive sampling will be used to recruit a comparable number of participants in terms of age, gender, length of stay in Canada, and sponsorship status. We will include 6 to 8 participants per group session and hold 2 to 4 group sessions with each category of participants within each immigrant community. The resulting sample (size) will be adequate for subgroup analyses, while ensuring feasibility. The subgroup analysis will compare participants’ responses by gender (32 women and 32 men per immigrant community), immigrant community (128 older adults per community), and city (64 per city). These subgroups’ sizes exceed the number recommended to reach information saturation in qualitative data analysis (which is usually 20 to 25 participants) [58] and provide statistical power to detect medium-sized differences, setting power at .80 and *P* at .05 [59]. For family members’ stakeholder group, separate sessions will be held for daughters or daughters-in-law (1-2 sessions per city), sons or sons-in-law (1-2 sessions per city), and grandchildren (1-2 sessions per city; 48 family members in total). We will attempt to represent diversity in terms of length of stay in Canada, income, employment, having (or not) children who live at home, having (or not) an older adult living with them, and extended family coresidence. Informal and formal community leaders will include leaders working with, providing support for, and/or advocating on behalf of older adults in their community (eg, faith leaders, media figures, and community advocates) and social, settlement, and health care workers from each community. One focus group session will be held with (6 to 8) community leaders in each city. One focus group session will be held in each city with (6 to 8) service providers (ie, social, settlement, and health), who work with older immigrants but do not belong to the selected immigrant communities.

Before data analysis, the audio recordings of the focus groups conducted in the 3 languages will be transcribed and then translated into English by the research assistants who conducted the sessions. Translations will be verified by our community partners fluent in the respective language. Researchers will independently code transcripts and reach consensus on these at regular meetings; they will also compare the codes to generate subcategories that reflect commonalities and differences in perceptions of social support and services within and across subgroups and communities. Gaps in emerging subcategories will be addressed in subsequent focus groups. The analysis will explore social dimensions (eg, gender, culture, language, length of stay in the country, and extended family coresidence); actual or preferred size and composition (gender, culture, age, and location) of informal networks; and types and frequency of support, reciprocity, conflict, and formal social support services (eg, police, legal, employment, continuing education classes, housing, and transportation). The data will be analyzed at different levels, beginning with the group session and then integrated across subgroups, communities, and the 4 cities to reveal common and unique issues. Member checks, peer debriefing, gathering diverse perspectives, careful documentation of the analysis procedures, identification and verification of themes, and interpretation of the findings will ensure the trustworthiness of the results [57].

*Phase 2* will involve examining gaps between the need for age-friendly formal social support services and the availability of these services in each community and city, especially spatial equity in service provision and utilization. A geographical or spatial analysis approach using a geographic information system (GIS) will clarify individual travel behavior in accessing services among older immigrants and identify location gaps. This analysis will include 2 stages. The first stage (stage 1) will use qualitative GIS findings [59,60] to visualize and analyze patterns in using and accessing senior services, such as housing, transportation, employment, and continuing education classes, based on information collected from focus groups in phase 1. The second stage (stage 2) will apply various accessibility models to systematically examine the spatial relationship between residential patterns of older immigrants and the distribution of services for older adults, in terms of service capacity, service language, types of service offered, etc at each site.

A combination of qualitative and quantitative datasets will be utilized in both stages: information from focus groups (eg, location of participants and services used, frequency of visits, perception of service providers, and quality of social networks), 2016 Census data about neighborhood sociodemographic characteristics (eg, proportion of older adults), and data about services for older adults (eg, service provider locations and attributes) gathered from various sources such as Ontario 211 and municipal websites (eg, settlement.org).

Specifically, stage 1 will use GIS data to explore travel patterns in accessing services and how social networks, individual characteristics, and service availability affect activity space [61]. Residential locations of focus group participants will be geocoded, and a simple frame of activity space will be created for each individual, including the locations of the service provider(s) they visit and the locations and durations of other activities they engage in immediately before or after (eg, grocery shopping and visiting a friend) these visits. We will calculate the distance each participant travels to access services and compare travel patterns and extent of activity space with the participant’s quality of social network, their perceptions about service provider(s) for older immigrants, and other basic demographic characteristics. Finally, we will compare the constructed activity space with the distribution of all service providers using the master dataset of senior services. This will help reveal some of the complex spatial relationships between residential location, neighborhood resource distribution, personal characteristics, and social network. For example, the results will help clarify whether participants would choose to bypass the closest service location in favor of an inconveniently located one that has language and culturally appropriate services or whether clustering services for older immigrants and other coethnic resources (eg, church and grocery stores) might encourage utilization.

Stage 2 will involve a systematic evaluation of service gaps for older adults. Census data have some limitations, preventing comparison between older adults’ places of birth, age, location, and country of origin. Therefore, we will clarify service gaps for the older adult population in each study area as a whole using 3 different spatial analysis techniques and models. First, we will analyze service areas (the catchment area of each provider) considering 3 modes of transportation: walking, public transit, and private vehicle. Each service area will be decided empirically based on the travel behavior reported by focus group participants and insights generated from focus groups with service providers. Second, we will use the cumulative accessibility model as simple measures of accessibility from each census tract to service locations for older adults. This model can calculate the number of services included within the travel threshold from each census tract centroid. Finally, we will use an advanced accessibility model (2-step floating catchment area model) to compute spatial accessibility to service providers by considering the spatial distribution of services and competition among service users in census tracts [62,63].

Together, the results of these analyses will provide important insights about areas of underservice for older adults and possibly misdistribution of existing service providers. We will assign access scores to each census tract, which will have important policy implications for accessing the efficiency of existing services and programs for older adults. Although these spatial analyses will be performed based on census data containing the entire older adult population, by overlaying the accessibility maps on the distribution of study populations, we will provide rich data about the relationships between group-specific residential patterns and service accessibility. The data collected during focus groups about individual travel behavior will be critically important to determine the travel threshold parameters. Most previous research has used hypothetical threshold and catchment size [64,65]. Therefore, the integration of qualitative (focus groups) and quantitative (spatial accessibility models) data in Stages 1 and 2 is particularly innovative and advantageous.

### Ethics Approval and Consent to Participate

This study has received approval from the Research Ethics Boards of Ryerson University in Toronto, Ontario; King’s University College at Western University in London, Ontario; the University of Ottawa in Ottawa, Ontario; and Wilfred Laurier University in Waterloo, Ontario. Participants will be informed that their participation in the study is completely voluntary, and they can choose whether to be in the study or not. Participants will review and sign informed consent, in English or their own language (based on their preference), before participating. If any participant appears to be needing help, the moderator will provide supportive listening and information on how to access suitable agencies or services as needed. Participants will have the option to leave the group discussion at any time for any reason. If, after participation in a group discussion, participants decide they no longer want to be part of the study, they can choose to exclude data collected from them. For this to occur, the participant must inform the research team at the end of the interview or within 8 weeks after joining the study. Withdrawal within this time will result in removal and destruction of data contributed.

## Results

Quantitative and qualitative data collection is ongoing. To date, focus groups with older women and men, and family members for each community at each site have been completed. In addition, the datasets for the GIS analysis have been secured and are currently being cleaned.

## Discussion

### Overview

Over the last 20 years, more immigrants have settled in suburban areas [66] instead of downtown cores. However, this change has not been reflected in funding, service, and resource allocation, resulting in extensive unmet social support needs [67]. Considerable research has focused on how place affects well-being [60-62,68-74] and the need for community locations where newcomers can build social networks and participate in cultural and political life [75,76]. However, little is known from a comparative perspective about how older immigrants access and use such social networks in small, midsized, and large cities. This study will compare less-established immigrant communities (with little or no support within their own community) with better-established communities (with more internal support) in large, medium, and small cities to help clarify key settlement and integration outcomes in the context of aging well. Specifically, there is limited knowledge on the extent to which older immigrants access and engage in formal and informal social activities as well as on their social needs, nature and composition of their actual and preferred social networks, and use of formal and informal supports. This research has also generally involved a single disciplinary perspective. Our multidisciplinary study will examine social needs, networks, and supports among older immigrants in a variety of geographical settings to identify factors that affect their access to and use of informal and formal social supports and the gaps between service needs and provision. The findings will advance scholarship in social work, immigration studies, nursing, and geography and will inform policy debates and practice change at local, national, and possibly international levels.

### Strengths and Limitations

Recruitment of older adults from different immigrant communities is a potential challenge that will be mitigated by our collaborative and participatory approach to research. In addition, to fully clarify the factors (social, cultural, economic, and geographic) that affect the ability of older immigrants to access social networks and support, we have also brought together community partners from a range of sectors. We plan to develop evidence-based ways to promote social connection, reduce social isolation, and improve well-being among older immigrants living in large, medium, and small cities. We have already confirmed the participation of community partner organizations in the 4 cities: each will facilitate the research activity with the shared goal of advocating for and supporting the needs of older immigrants. Each has proven capacity in offering services to immigrants. These organizations rely on evidence-based results to inform their ongoing programs and the design, implementation, and evaluation of tools.

### Conclusions

The findings will benefit the organizations serving older immigrants greatly and will also lead to social and cultural benefits for the communities represented by the organizations and older immigrants, in particular. The proposed partnership will develop evidence-based ways to promote social connections and reduce social isolation to make communities and cities more supportive of older immigrants.

## Abbreviations

GIS: Geographic Information System
